# Understanding Decision-Making in the Adoption of Digital Health Technology: The Role of Behavioral Economics’ Prospect Theory

**DOI:** 10.2196/32714

**Published:** 2022-02-07

**Authors:** Waqas Ullah Khan, Aviv Shachak, Emily Seto

**Affiliations:** 1 Department of Health Informatics Institute of Health Policy, Management and Evaluation University of Toronto Toronto, ON Canada; 2 Department of Psychiatry University Hospital Limerick Limerick Ireland

**Keywords:** decision-making, digital health technology adoption, prospect theory

## Abstract

The decision to accept or reject new digital health technologies remains an ongoing challenge among health care patients, providers, technology companies, and policymakers. Over the past few decades, interest in understanding the choice to adopt technology has led to the development of numerous theories and models. In 1979, however, psychologists Kahneman and Tversky published their seminal research article that has pioneered the field of behavioral economics. They named their model the prospect theory and used it to explain decision-making behaviors under conditions of risk and uncertainty as well as to provide an understanding of why individuals may make irrational or inconsistent choices. Although the prospect theory has been used to explain decision-making in economics, law, political science, and clinically, at the individual level, its application to understanding choice in the adoption of digital health technology has not been explored. Herein, we discuss how the main components of the prospect theory’s editing phase (framing effect) and evaluation phase (value function and weighting function) can provide valuable insight on why health care patients, providers, technology companies, and policymakers may decide to accept or reject digital health technologies.

## The Challenge to Accept or Reject a Digital Health Technology

Digital health technology includes a broad spectrum of categories, such as mobile health, clinical information systems, wearable devices, telehealth, and personalized medicine. From mobile fitness applications to machine learning technologies used to improve disease diagnosis, clinical decision-making, and health care delivery, digital health is revolutionizing the field of medicine [1]. Nevertheless, the decision to accept or reject new digital health technologies remains an ongoing challenge among health care patients, providers, technology companies, and policymakers. Over the past few decades, interest in understanding the choice to adopt technology has led to the development of numerous theories and models.

## The Technology Acceptance Model

The Technology Acceptance Model (TAM), proposed by Fred Davis in 1989, is one of the most prominent theories developed to explain the behavioral intention to use a technological innovation [2]. Applying concepts from the theory of reasoned action and theory of planned behavior, the TAM suggests that the intended use of technology is determined by its “perceived ease of use” and “perceived usefulness.” Both factors are connected with one’s behavioral intention, which is linked to actual behavior and system characteristics (external variables) [2,3]. Although the TAM’s simplified rationale has made it popular among researchers, academics, and within the technology industry, it is also one of its main criticisms [4]. In many studies, it is reduced to concepts that make outcomes such as intended use the primary finding, which lowers the TAM’s explanatory capability and provides little insight on the actual use of the technology studied [4].

## The Prospect Theory

In 1979, however, psychologists Kahneman and Tversky published their seminal research article that has pioneered the field of behavioral economics by providing a novel understanding of decision-making. They named their model the prospect theory and used it to explain decision-making behaviors under conditions of risk and uncertainty [5]. This theory also provides an understanding of why individuals may make irrational or inconsistent decisions [5]. Although the prospect theory has been used to explain decision-making in economics, law, political science, and clinically, from the physician’s perspective, its application to understanding choice in the adoption of digital health technology has not been explored [6]. Notably, this can provide valuable insight on why health care patients, providers, technology companies, and policymakers may decide to accept or reject digital health technologies under conditions of risk or uncertainty as well as provide an explanation for the perceived irrational or inconsistent decisions concerning the adoption of digital health technology in medicine [7].

## The Editing Phase of the Prospect Theory

The prospect theory consists of two main phases, an editing phase and an evaluation phase. In the editing phase, individuals perform a preliminary analysis of choices or prospects they are provided. This allows one to organize and edit the choices available with the aim of simplifying the decision-making process [5]. It is also during this phase that reference points, often referred to as framing effects, are made to help guide one’s decision. However, framing effects can be influenced by the order, method, or wording in which they are presented [5].

Regarding the adoption of digital health technology, a framing effect can occur when a patient is offered a choice concerning their management of care. For example, individuals with a high risk of experiencing an acute cardiac event can be offered the choice to continue their current level of care (eg, routine clinic visits) or adopt a novel wearable digital health device, such as a smart watch that monitors their vital signs dynamically and advises them when to modify their lifestyle behaviors or seek medical attention.

Similar to Tversky and Kahneman’s framing effect experiments involving hypothetical infectious disease management scenarios, the adoption of wearable digital health devices can be framed in various manners. For instance, the use of a wearable digital health device can be described as providing a 100% likelihood of improving one-third of users’ quality of life when compared with the current standard of care. Conversely, the same device can be described as offering a 100% probability that two-thirds of users will not experience any quality-of-life improvement when compared with the current standard of care. In the first scenario, the choice is framed as an opportunity to achieve a gain of improved quality of life. Conversely, choosing the digital health device in the second example is framed as a loss as there is a greater likelihood of no benefit, but wasted time. In reality, the outcomes of both choices are the same. However, Kahneman and Tversky discovered that people make different decisions among the same choices with certainty in gains (100% likelihood of improving one-third of users’ quality of life) being more desirable than certainty in loses (100% probability that two-thirds of users do not experience an improvement in their quality of life) [5].

## The Evaluation Phase of the Prospect Theory

Once a choice is simplified and framed for a decision, the evaluation phase of the prospect theory begins. There are two key components in this phase, the value function and weighting function. The value function refers to the original reference point or choice, which is often the individual’s current status. This baseline reference point is then compared with an operative reference point, often a future goal. An important characteristic of the value function is that the worth of each choice is dependent on how the individual perceives the change between their current and future states [5]. For example, when compared with the current standard of care (routine clinic visits), the value function for patients with cardiovascular disease using a digital health smart watch can be described as providing continuous medical feedback that fosters patient autonomy, education, and personalized preventative care while reducing health care costs.

Another important aspect of the value function is that individuals are more risk averse in the context of making gains. For example, many telehealth companies are reporting significant increases in revenue as their digital platform usage grows due to the United States’ COVID-19 government policies promoting social distancing, equal reimbursement for virtual and physical visits, and the permission granted to physicians to use these tools to practice care across state lines, as well as without requiring additional state medical licenses. If these companies choose to save the additional profits or gains made during the COVID-19 pandemic instead of using them to upgrade or develop new products, they are demonstrating the value function’s risk aversion approach. Conversely, Kahneman and Tversky found that individuals are more likely to take risks in the context of experiencing losses [5]. This would mean that if telehealth companies experienced substantial revenue losses during the COVID-19 pandemic, they would likely take greater financial risks to return to a favorable position [5].

The final concept of the value function is known as loss aversion, which states losses are more painful than equal gains are pleasing. In a hypothetical scenario, health care policymakers are presented with a choice to adopt a novel electronic health record (EHR) system that aims to reduce hospital costs and inefficiencies by improving resource management. However, the risk in adopting this system is a loss of $100 million of taxpayers’ money if it fails. Conversely, if it succeeds, $100 million of taxpayers’ money will be saved. In their research, Kahneman and Tversky found that when individuals are presented with a similar loss or equal gains choice, they must be offered at least twice as much in gains as compared with losses to take the risk [8]. Concerning the EHR example above, this means health care policymakers would be more likely to adopt the system if the net gain or money saved is $200 million or more when compared with the risk of losing $100 million if it failed.

Regarding the evaluation phase’s weighting function, Kahneman and Tversky showed that people give higher value to outcomes that are less likely to develop, while giving less importance to outcomes with a medium to high probability of occurring. Moreover, individuals are more likely to take a sure loss against a small possibility of a larger loss. This is often seen with the purchase of warranties or insurance, a sure loss, on digital health devices such as smart watches that monitor vital signs or EHR systems. In this situation, the cost of repairing the device if it breaks is higher (a small possibility of a larger loss) when compared with the minimal loss of insuring the product (a sure loss).

## Conclusion

The prospect theory, as illustrated by the examples given, provides valuable insight on why health care patients, providers, technology companies, and policymakers may decide to accept or reject digital health technologies. Given the rapid growth of this industry, the medical field and its stakeholders have more choice than ever regarding the adoption of digital health tools. Nevertheless, by understanding why choices are made, we can make better informed decisions when selecting our next digital health technology prospect.

## Abbreviations

EHR: electronic health record
TAM: Technology Acceptance Model
